# Distinctive antibody responses to *Mycobacterium tuberculosis* in pulmonary and brain infection

**DOI:** 10.1093/brain/awae066

**Published:** 2024-03-05

**Authors:** Marianna Spatola, Nadège Nziza, Edward B Irvine, Deniz Cizmeci, Wonyeong Jung, Le Hong Van, Le Thanh Hoang Nhat, Vu Thi Ngoc Ha, Nguyen Hoan Phu, Dang Trung Nghia Ho, Guy E Thwaites, Douglas A Lauffenburger, Sarah Fortune, Nguyen Thuy Thuong Thuong, Galit Alter

**Affiliations:** Ragon Institute of Mass General, MIT and Harvard, Cambridge, MA 02139, USA; Ragon Institute of Mass General, MIT and Harvard, Cambridge, MA 02139, USA; Ragon Institute of Mass General, MIT and Harvard, Cambridge, MA 02139, USA; Department of Immunology and Infectious Diseases, Harvard T.H. Chan School of Public Health, Boston, MA 02115, USA; Ragon Institute of Mass General, MIT and Harvard, Cambridge, MA 02139, USA; Department of Biological Engineering, Massachusetts Institute of Technology, Cambridge, MA 02139, USA; Ragon Institute of Mass General, MIT and Harvard, Cambridge, MA 02139, USA; Department of Biological Engineering, Massachusetts Institute of Technology, Cambridge, MA 02139, USA; Oxford University Clinical Research Unit, Centre for Tropical Medicine, 700000 Ho Chi Minh City, Vietnam; Oxford University Clinical Research Unit, Centre for Tropical Medicine, 700000 Ho Chi Minh City, Vietnam; Oxford University Clinical Research Unit, Centre for Tropical Medicine, 700000 Ho Chi Minh City, Vietnam; Oxford University Clinical Research Unit, Centre for Tropical Medicine, 700000 Ho Chi Minh City, Vietnam; Vietnam National University, School of Medicine, 700000 Ho Chi Minh City, Vietnam; Hospital for Tropical Diseases, 700000 Ho Chi Minh City, Vietnam; Pham Ngoc Thach University of Medicine, 700000 Ho Chi Minh City, Vietnam; Oxford University Clinical Research Unit, Centre for Tropical Medicine, 700000 Ho Chi Minh City, Vietnam; Centre for Tropical Medicine and Global Health, Nuffield Department of Medicine, Oxford University, Oxford, OX3 7LG, UK; Department of Biological Engineering, Massachusetts Institute of Technology, Cambridge, MA 02139, USA; Department of Immunology and Infectious Diseases, Harvard T.H. Chan School of Public Health, Boston, MA 02115, USA; Oxford University Clinical Research Unit, Centre for Tropical Medicine, 700000 Ho Chi Minh City, Vietnam; Centre for Tropical Medicine and Global Health, Nuffield Department of Medicine, Oxford University, Oxford, OX3 7LG, UK; Ragon Institute of Mass General, MIT and Harvard, Cambridge, MA 02139, USA

**Keywords:** TB meningitis, neuroinflammation, Fc receptors, antibody-mediated phagocytosis, antibody-mediated complement deposition

## Abstract

*Mycobacterium tuberculosis*, the causative agent of tuberculosis (TB), remains a global health burden. While *M. tuberculosis* is primarily a respiratory pathogen, it can spread to other organs, including the brain and meninges, causing TB meningitis (TBM). However, little is known about the immunological mechanisms that lead to differential disease across organs. Attention has focused on differences in T cell responses in the control of *M. tuberculosis* in the lungs, but emerging data point to a role for antibodies, as both biomarkers of disease control and as antimicrobial molecules. Given an increasing appreciation for compartmentalized antibody responses across the blood–brain barrier, here we characterized the antibody profiles across the blood and brain compartments in TBM and determined whether *M. tuberculosis*-specific humoral immune responses differed between *M. tuberculosis* infection of the lung (pulmonary TB) and TBM.

Using a high throughput systems serology approach, we deeply profiled the antibody responses against 10 different *M. tuberculosis* antigens, including lipoarabinomannan (LAM) and purified protein derivative (PPD), in HIV-negative adults with pulmonary TB (*n* = 10) versus TBM (*n* = 60). Antibody studies included analysis of immunoglobulin isotypes (IgG, IgM, IgA) and subclass levels (IgG1–4) and the capacity of *M. tuberculosis*-specific antibodies to bind to Fc receptors or C1q and to activate innate immune effector functions (complement and natural killer cell activation; monocyte or neutrophil phagocytosis). Machine learning methods were applied to characterize serum and CSF responses in TBM, identify prognostic factors associated with disease severity, and define the key antibody features that distinguish TBM from pulmonary TB.

In individuals with TBM, we identified CSF-specific antibody profiles that marked a unique and compartmentalized humoral response against *M. tuberculosis*, characterized by an enrichment of *M. tuberculosis*-specific antibodies able to robustly activate complement and drive phagocytosis by monocytes and neutrophils, all of which were associated with milder TBM severity at presentation. Moreover, individuals with TBM exhibited *M. tuberculosis*-specific antibodies in the serum with an increased capacity to activate phagocytosis by monocytes, compared with individuals with pulmonary TB, despite having lower IgG titres and Fcγ receptor-binding capacity.

Collectively, these data point to functionally divergent humoral responses depending on the site of infection (i.e. lungs versus brain) and demonstrate a highly compartmentalized *M. tuberculosis*-specific antibody response within the CSF in TBM. Moreover, our results suggest that phagocytosis- and complement-mediating antibodies may promote attenuated neuropathology and milder TBM disease.

## Teaser

*Mycobacterium tuberculosis* causes pulmonary tuberculosis and tuberculosis meningitis, but the mechanisms by which the latter develops are unknown. Spatola *et al.* show that antibodies against *Mycobacterium tuberculosis* in the brain may play a role in the development and severity of tuberculosis meningitis.

## Introduction

Infection with *Mycobacterium tuberculosis* (*Mtb*), the causative agent of tuberculosis (TB), remains the leading cause of death due to a single infectious disease, with one third of the global population infected or persistently exposed, greater than 10.6 million new infections annually and more than 1.5 million deaths in 2021 alone.^1^ Although the primary site of *Mtb* infection is in the lungs, *Mtb* can spread to several other tissues and organs, including the brain and meninges, causing TB meningitis (TBM). Despite representing only 1%–5% of all *Mtb* infections worldwide,^2,3^ TBM is the most severe form of extrapulmonary TB disease. Even when promptly diagnosed and treated with appropriate antibiotics, TBM is associated with a high mortality (20%–50%), while survivors are at high risk of CNS complications and life-long neurological sequelae.^4^ Moreover, children and people living with HIV are at the highest risk of developing TBM, which can be particularly life-threatening within these vulnerable populations.^5,6^ Clinically, TBM manifestations closely resemble the presentation of other bacterial, viral or fungal CNS infections and non-infectious neuroinflammatory diseases, which renders the accurate diagnosis of TBM a significant challenge.^4,7^ Thus, there is an urgent need to better understand the pathogenesis that contributes to the evolution and poor outcomes of TBM, as well as to develop novel diagnostics able to identify TBM more reliably.

While a number of studies have probed the correlates of immune protection in pulmonary TB,^8,9^ less is known about the immunological signatures and mechanisms underlying *Mtb* infection of the CNS.^7^ Given the immune vulnerability of HIV-positive people to TBM, several studies have suggested that T cells, which are selectively depleted in the setting of HIV, likely play a key role in the control of *Mtb* within the brain.^10^ In contrast, the profile and functional role of *Mtb*-specific antibodies in the setting of TBM remain largely unexplored. Thus, here we leveraged high-throughput systems serology antibody profiling to comprehensively define *Mtb*-specific antibody responses across HIV-negative individuals with pulmonary TB and TBM. We hypothesized that examining humoral immune responses across these distinct manifestations of TB disease (pulmonary TB and TBM) and physiological compartments (serum and CSF) would reveal humoral signatures that mark the diverging disease states and provide mechanistic insights into the functional roles antibodies may play during TBM.

Distinct *Mtb*-specific humoral immune responses were observed in the serum and CSF from individuals with TBM, marked by enhanced titres in the peripheral circulation and highly specialized functions in the CSF. In particular, increased complement and neutrophil mediated opsinophagocytic activity was associated with milder TBM severity at presentation, pointing to specific CNS specific antibody mediated functions that may be key to clearance of the bacteria during brain infection. Overall, these data point to a highly compartmentalized *Mtb*-specific humoral immune response across the serum and CSF in TBM that differs from pulmonary TB disease and associates with disease severity.

## Materials and methods

### Study population and biological samples

Samples from 70 HIV-negative individuals with *Mtb* infection were enrolled in this study (Table 1), including 60 individuals with definite or probable TB meningitis (defined as ‘TBM’)^11^ and 10 with positive sputum *Mtb* pulmonary infection (defined as ‘pulmonary TB’), from a cohort of adults from Ho Chi Minh city, Vietnam, where TB infection is endemic (prevalence of 176 cases/100 000 inhabitants in 2020 according to the World Health Organization).^12^

**Table 1:** Clinical and paraclinical features, disease severity and mortality of patients with tuberculosis (TB) meningitis, pulmonary TB or non-infectious CNS disorders

Variable	Definite TBM *N* = 30	Probable TBM *N* = 25	Pulmonary TB *N* = 10	Non-infectious CNS disorders *N* = 10
Age, years, median (IQR)	30 (24, 41)	43 (29, 54)	45 (34, 50)	28 (26, 36)
Male, *n* (%)	19 (54%)	16 (64%)	9 (90%)	6 (67%)
HIV positive, *n* (%)	0 (0%)	0 (0%)	0 (0%)	0 (0%)
Microbiological tests^a^				
MGIT culture positive, *n* (%)	24 (69)	0 (0)	10 (100)	–
Xpert positive, *n* (%)	23 (66)	0 (0)	10 (100)	–
Smear positive, *n* (%)	32 (91)	0 (0)	–	–
CSF parameters				
Leucocytes, ×10^3^ cells/ml, median (IQR)	177 (91, 302)	144 (58, 264)	–	15 (8, 18)
Lymphocytes, %	61 (35, 88)	93 (90, 99)	–	87 (70, 91)
Neutrophils, %	34 (12, 65)	6 (2, 10)	–	13 (10, 30)
Proteins, g/l	1.54 (0.99, 2.32)	1.07 (0.60, 1.79)	–	0.35 (0.26, 0.56)
Glucose, mmol/l	1.80 (1.32, 2.56)	2.20 (1.50, 3.20)	–	4.11 (3.29, 4.21)
Lactate, mmol/l	5.81 (4.31, 7.63)	3.70 (3.30, 5.28)	–	2.28 (1.94, 2.98)
CSF/blood protein ratio	0.02 (0.01, 0.04)	0.02 (0.01, 0.03)	–	–
Blood parameters				
Leucocytes, ×10^6^ cells/m, median (IQR)	11.1 (9.4, 14.9)	8.8 (7.7, 10.8)	11.4 (10.0, 12.8)	11.7 (9.5, 14.6)
Lymphocytes, %	10 (6, 14)	12 (9, 19)	11 (9, 15)	13 (9, 14)
Monocytes, %	–	–	11.10 (10.65, 12.70)	–
Neutrophils, %	81 (80, 89)	77 (66, 82)	77 (73, 77)	81 (77, 83)
Eosinophils, %	–	–	–	0.54 (0.30, 1.20)
Platelets, g/l	292 (253, 344)	248 (214, 326)	–	258 (199, 301)
Protein, g/l	70 (64, 74)	71 (61, 73)	77 (76, 81)	–
Glasgow Coma Scale, median (IQR)	14 (9, 15)	14 (14, 15)	–	10 (9, 11)
BMRC^b^ TBM grading, *n*				
Mild (Stage I)	10	8	–	–
Moderate (Stage II)	10	9	–	–
Severe (Stage III)	10	8	–	–
Death, *n* (%)	5 (14%)	5 (20%)	0 (0%)	–

IQR = interquartile range; MGIT = Mycobacteria Growth Indicator Tube; TBM = TB meningitis.

^a^Microbiological tests were performed on CSF samples for TBM and sputum samples for pulmonary TB.

^b^British Medical Research Council (BMRC) TBM grading.

TBM diagnosis was established based on suggestive clinical presentation, MRI findings, CSF analyses and published criteria.^13,14^ ‘Definite TBM’ diagnosis (*n* = 30) was established if *Mtb* could be identified in CSF using three different techniques (smear, GeneXpert and culture), whereas a ‘probable TBM’ diagnosis (*n* = 25) was considered when clinical, radiological and CSF criteria were met, but *Mtb* was not isolated from CSF using any of the above-mentioned techniques. Clinical severity of individuals with TBM was measured using the modified British Medical Research Council (BMRC) TBM grading system, which categorizes patients into three grades with increasing order of severity: mild (or Stage I, with Glasgow Coma Scale of 15 without focal neurological deficits); moderate (or Stage II, with Glasgow Coma Scale of 14 to 11 and focal neurological deficits); and severe (or Stage III, with Glasgow Coma Scale of ≤ 10, with or without focal neurological deficits). A similar number of individuals (*P* = 0.98) per severity group was selected in both definite (*n* = 30) and probable (*n* = 25) TBM cohorts. Microbiological tests for confirmation of *Mtb*, clinical and paraclinical features, disease severity and mortality are described in Table 1.

Serum samples were available from individuals with pulmonary TB, paired serum and CSF samples were available from individuals with TBM. All samples were obtained before antibiotic treatment. CSF from individuals with non-infectious CNS disorders (brain tumour, *n* = 2; cerebral haemorrhage, *n* = 1; autoimmune encephalitis, *n* = 4; psychiatric disorder, *n* = 1; seizure disorder, *n* = 2; Table 1) from the same endemic area were used as negative controls. In compliance with the Declaration of Helsinki, written informed consent was obtained from each participant or an accompanying relative if the participant was unable to provide consent. The study protocol and informed consent form were approved by the institutional review board of the Hospital for Tropical Diseases, Vietnam and the Oxford Tropical Research Ethics Committee, UK.

### Antigens

The *Mtb*-specific antibody response was assessed against *Mtb*-lysate derived purified protein derivative (PPD; Statens Serum Institute), as well as against the following *Mtb*-antigens known to be robust targets of antibodies^9,15,16^: Lipoarabinomannan (LAM; BEI Resources, NR-14848), PstS1 (BEI Resources, NR-14859), Apa (BEI Resources, NR-14862) and HspX, ESAT/CFP10, Ag85 A/B, Mpt46 and GroES (all provided by T. Ottenhoff and K. Franken, prepared as described previously^17,18^). Haemagglutinin (HA|) from two strains of Influenza virus A (ImmuneTech IT-003-SW12p and ImmuneTech IT-003-001p) was used as positive control and PBS levels were used as a negative control.

### Antibody subclass, isotype and Fc-receptor binding

Total IgG were calculated using an ELISA Human IgG kit (Invitrogen 88–50550-088), following the manufacturer’s instructions.

Antigen-specific antibody subclass and isotype levels, in addition to Fc-receptor (FcR) binding, were measured in serum and CSF samples using a customized multiplexed Luminex assay as previously reported.^19^ Briefly, each antigen was coupled to different magnetic Luminex beads by carbodiimide-NHS ester coupling. Serum (dilution 1:100) or CSF (dilution 1:10) samples were then added to the beads to form immune complexes. After an incubation for 2 h at room temperature (RT), immune complexes were washed, and subclasses and isotypes were detected using phycoerythrin (PE)-conjugated mouse anti-human IgG1, IgG2, IgG3, IgG4, IgA1, IgA2 or IgM (Southern Biotech) at 1.3 μg/ml. Concerning FcRs, they were purchased from Duke Human Vaccine Institute, and binding capacity to Avi-tagged FcγR2A, FcγR2B, FcγR3A, FcγR3B and FcαR was assessed using PE-streptavidin (Agilent Technologies) that was coupled to the different FcRs as previously described.^20,21^ After 1 h of incubation with subclasses/isotypes or FcRs, immune complexes were washed and median fluorescence intensity (MFI) was determined on an iQue analyzer (Intellicyt).

### Antibody-dependent functional assays

#### Antigen biotinylation

All antibody-dependent functional assays were assessed for PPD- and LAM-specific antibodies. PPD was biotinylated using NHS-Sulfo-Biotin (#21338, Thermo). Instead, as LAM is a glycolipid, biotinylation protocol differed from that of PPD. As previously described,^9^ for every 100 μg of LAM (dissolved in dH2O), 10 μl of 1 M sodium acetate (NaOAc) and 2.2 μl of 50 mM sodium periodate (NaIO_^4^_) were added. This oxidation reaction proceeded for 45–60 min on ice in the dark. Next, 12 μl of 0.8 M NaIO_4_ was added to block oxidation, and the solution was incubated for 5 min at RT in the dark. The oxidized LAM was then transferred to a new tube, and 10 μl of 1 M NaOAc and 22 μl of 50 mM hydrazide biotin (Sigma) were added. This biotinylation reaction proceeded for 2 h at RT. Excess biotin from both PPD and LAM preparations was then removed using Amicon Ultra 0.5-ml columns (3K, Millipore Sigma) according to the manufacturer’s instructions.

#### Antibody-dependent cellular phagocytosis

For antibody-dependent cellular phagocytosis (ADCP), we determined the capacity of PPD-specific and LAM-specific antibodies to activate THP-1 monocyte phagocytosis using a bead-based phagocytic assay as previously described.^9,21^ Biotinylated LAM or PPD were coupled to FITC-conjugated neutravidin beads (Thermo Fisher F8776). LAM or PPD antigen-coated beads were incubated with either serum (dilution 1:500) or CSF (dilution 1:20) for 2 h at 37°C. After washing the immune complexes, THP-1 monocytes (0.25 M cells per well) were added and incubated overnight at 37°C. Cells were then fixed with 4% paraformaldehyde and analysed by flow cytometry. A phagocytosis score was calculated as the (percentage of bead-positive cells) × (MFI of bead-positive cells) divided by 100 000.

#### Antibody-dependent neutrophil phagocytosis

Similarly to ADCP, PPD- and LAM-specific antibody-dependent neutrophil phagocytosis (ADNP) was performed using a bead-based phagocytic assay as described previously.^9,21,22^ Briefly, after the formation of immune complexes composed of antigen-coupled neutravidin beads (Thermo Fisher F8776) and antibodies from serum (dilution 1:500) or CSF (dilution 1:20), human neutrophils obtained from healthy donors’ blood were added and incubated for 1 h at 37°C. Neutrophils were surface stained with anti-CD66b Pac blue antibody (BioLegend 305112), then fixed with 4% paraformaldehyde (PFA) and analysed by flow cytometry. Phagocytosis score was calculated as the (percentage of microsphere-positive CD66b cells) × (MFI of bead-positive cells) divided by 100 000.

#### Antibody-dependent complement deposition

PPD- and LAM-specific antibody-dependent complement deposition (ADCD) were performed as previously described.^23^ LAM was biotinylated as described for ADCP. In brief, biotinylated PPD or LAM were coupled to red fluorescent neutravidin microspheres (Thermo Fisher F8775), followed by an incubation with serum (dilution 1:40) or CSF (dilution 1:5) samples for 2 h at 37°C. Immune complexes were then washed and incubated with complement factors from guinea pig during 20 min at 37°C (Cedarlane CL4051). To stop the complement reaction, beads were washed with 15 mM EDTA. The deposition of complement was detected by 1:100 diluted fluorescein-conjugated goat IgG to guinea pig complement C3b (MP Biomed 855385), and relative C3 deposition was quantified by flow cytometry.

#### Antibody-dependent natural killer cell activation

An ELISA was performed to measure antibody-dependent natural killer cell activation (ADNKA) as previously described.^9,24^ Briefly, ELISA plates were coated with 150 ng per well of PPD or LAM, incubated overnight at 4°C; then serum (dilution 1:100) or CSF (dilution 1:10) samples were added for a 2-h incubation at 37°C. The day before adding the samples, NK cells were isolated from buffy coats, generated from healthy donors, using a RosetteSep NK enrichment kit (Stem Cell Technologies 15065) and were incubated overnight at 37°C with 1 ng/ml of interleukin-15 (IL-15). After the 2-h sample incubation, NK cells were added to the ELISA plates and stained with anti-CD107α (BD 555802) and treated with a protein-transport inhibitor (BD Bioscience 554724) and with brefeldin A (Sigma B7651). After a 5-h incubation at 37°C, surface cell staining was performed with anti-CD3 (BD 558117), anti-CD16 (BD 557758) and anti-CD56 (BD 557747) during a 20-min incubation at RT. NK cells were then fixed using Perm A and Perm B (Thermo Fisher GAS001S100) to allow intracellular staining with anti-IFNγ (BD 340449) and anti-MIP1β (BD 550078). NK cells were then analysed by flow cytometry and defined as CD3−CD56+ cells, and activation was identified as CD107a+IFNγ+CCL4+.

### Statistical and computational analysis

Univariate comparative analyses of antibody features between groups or across compartments (serum versus CSF) was performed using non-parametric tests (Mann–Whitney, Kruskal–Wallis, Friedman test) or parametric tests (paired *t*-test) as appropriate. Analyses were carried out using GraphPad Prism with Tukey corrections for multiple comparisons. Spearman’s correlation coefficients were calculated, and *P*-values were obtained for two-tailed tests.

Multivariate analyses were performed with R (version 4.1.0). Luminex data were log_10_-transformed. All data were *z*-scored. Missing values were imputed using R package ‘DMwR’. A partial least square discriminant analysis (PLSDA) with least absolute shrinkage selection operator (LASSO) was used to select antibody features that contributed most to the discrimination between pulmonary TB and TBM, as described.^25^ Antibody features were ranked according to their Variable Importance in Projection (VIP) score, for which higher values indicate higher contribution to the model, or by their first latent variable loadings (LV1). A 5-fold cross validation was performed to test model robustness. The model was validated by comparing it to a null model, for which the modelling approach was repeated 100× with random shuffling of labels. *P*-values were obtained from the tail probability of the accuracy distribution of the control model. This procedure was repeated for 10 replicates of cross-validation, and median *P*-values were reported.

An orthogonalized partial least squares regression (OPLSR) was used to mathematically identify the key antibody features contributing to variation in TBM severity (from mild to severe). The performance of the algorithm was evaluated with *R2* and *Q2* metrics. A LASSO was first used to select antibody features that best discriminated mild versus severe TBM (which were considered the most extreme clinical phenotypes and for which we expected to observe the most divergent antibody signatures). The 5-fold cross validation was performed as described for PLSDA.

## Results

### Detectable *Mtb*-specific antibodies in the CSF of TBM patients

**Figure 1:**
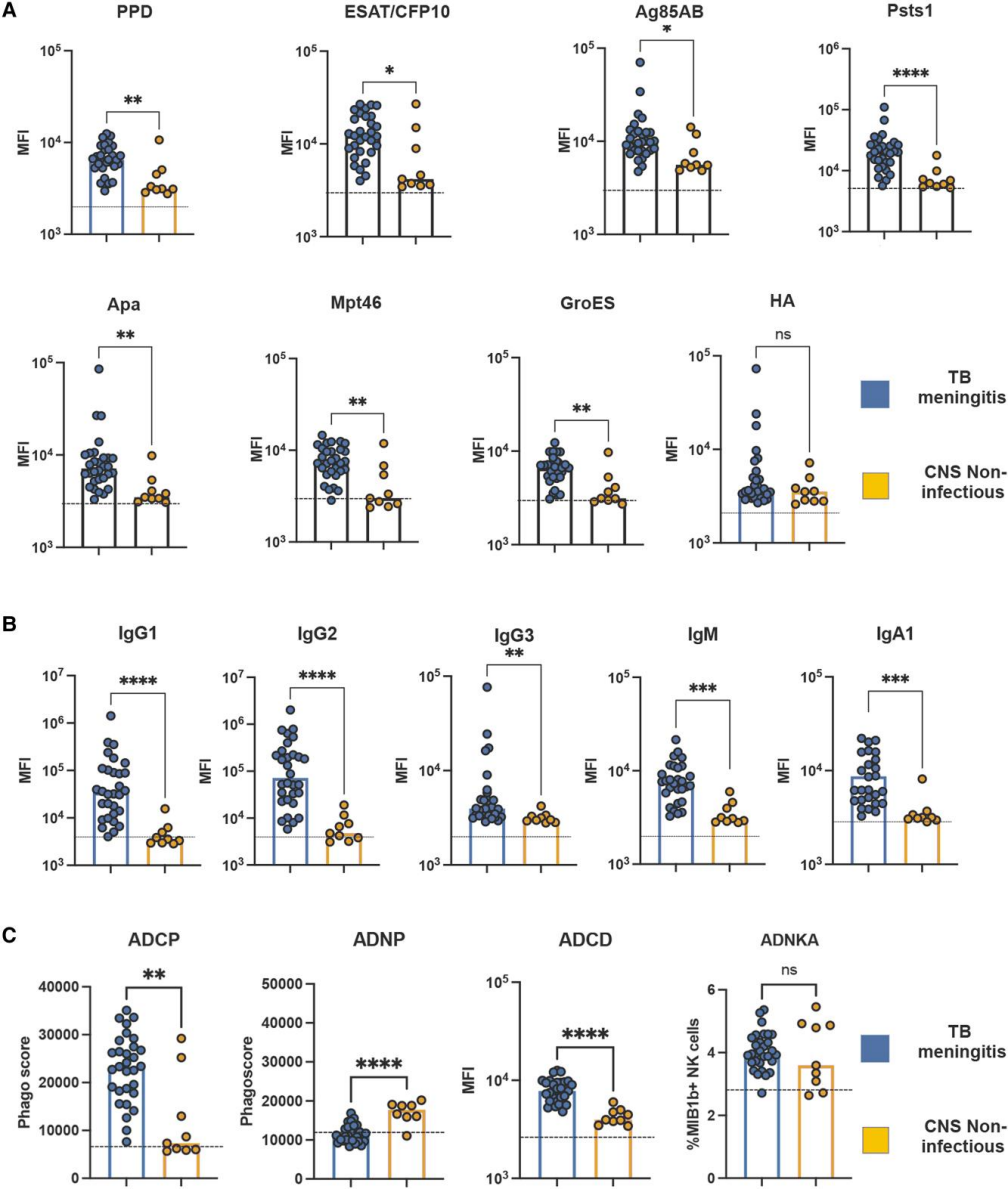
**High levels of functional and Fc-receptor binding *Mycobacterium tuberculosis*-specific antibodies in CSF from individuals with tuberculosis meningitis**. (**A**) Scatter-dot plot showing high levels of IgG1 antibodies against *Mtb* antigens [purified protein derivative (PPD), ESAT/CFP10, Ag85 A/B, Psts1, Apa, Mpt46 and GroES] in CSF from individuals with tuberculosis (TB) meningitis (*n* = 30) compared with non-infectious CNS disorders (*n* = 10). These differences are not observed in Flu-HA specific antibodies. (**B**) Scatter-dot plot showing lipoarabinomannan (LAM)-specific Ig classes (IgG, IgM, IgA), subclasses (IgG1–4) in TB meningitis (*n* = 30) compared with non-infectious CNS disorders (*n* = 10). LAM-specific antibodies from TB meningitis show higher IgG1, IgG2, IgM and IgA levels, compared with non-infectious CNS disorders. Each dot represents an individual. Bars represent median, dotted line indicates PBS level. Mann–Whitney statistics; **P < 0.01, ***P < 0.001, ****P < 0.0001. ADCD = antibody-dependent complement deposition; ADCP = antibody-dependent cellular phagocytosis; ADNKA = antibody-dependent natural killer cell activation; ADNP = antibody-dependent neutrophil phagocytosis; MFI = median fluorescence intensity.

We first aimed to determine whether *Mtb*-specific antibodies were detectable in the CSF of individuals with definite TBM (clinical information provided in Table 1). First, we determined the presence of IgG1 antibodies, the most abundant antibody in circulation, against several *Mtb* antigens. High levels of *Mtb-*specific IgG1 against PPD, ESAT/CFP10, Ag85A/B, PstS1, Apa, Mpt46 and GroES were observed in the CSF of nearly all TBM patients but not in control subjects with non-infectious CNS disorders from the same endemic region (Fig. 1A). Similar to the protein antigens, glycolipid-specific IgG1 antibodies to the most immunodominant surface glycan on *Mtb*, LAM, were significantly expanded in the CSF of individuals with TBM (Fig. 1B). Conversely, no difference in influenza HA-specific IgG1 levels in the CSF was noted across the groups (Fig. 1A).

Beyond IgG1 titres, the CSF of TBM patients was additionally enriched with a diverse subclass (IgG1, 2, 3, 4) and isotype (IgM and IgA) LAM-specific response in the setting of TBM (Fig. 1B). These data indicate that, despite the highly endemic nature of TB in Vietnam, *Mtb*-specific antibodies were specifically enriched in the CSF of individuals with TBM.

### Distinct serum and CSF *Mtb*-specific antibody profiles in TBM

**Figure 2:**
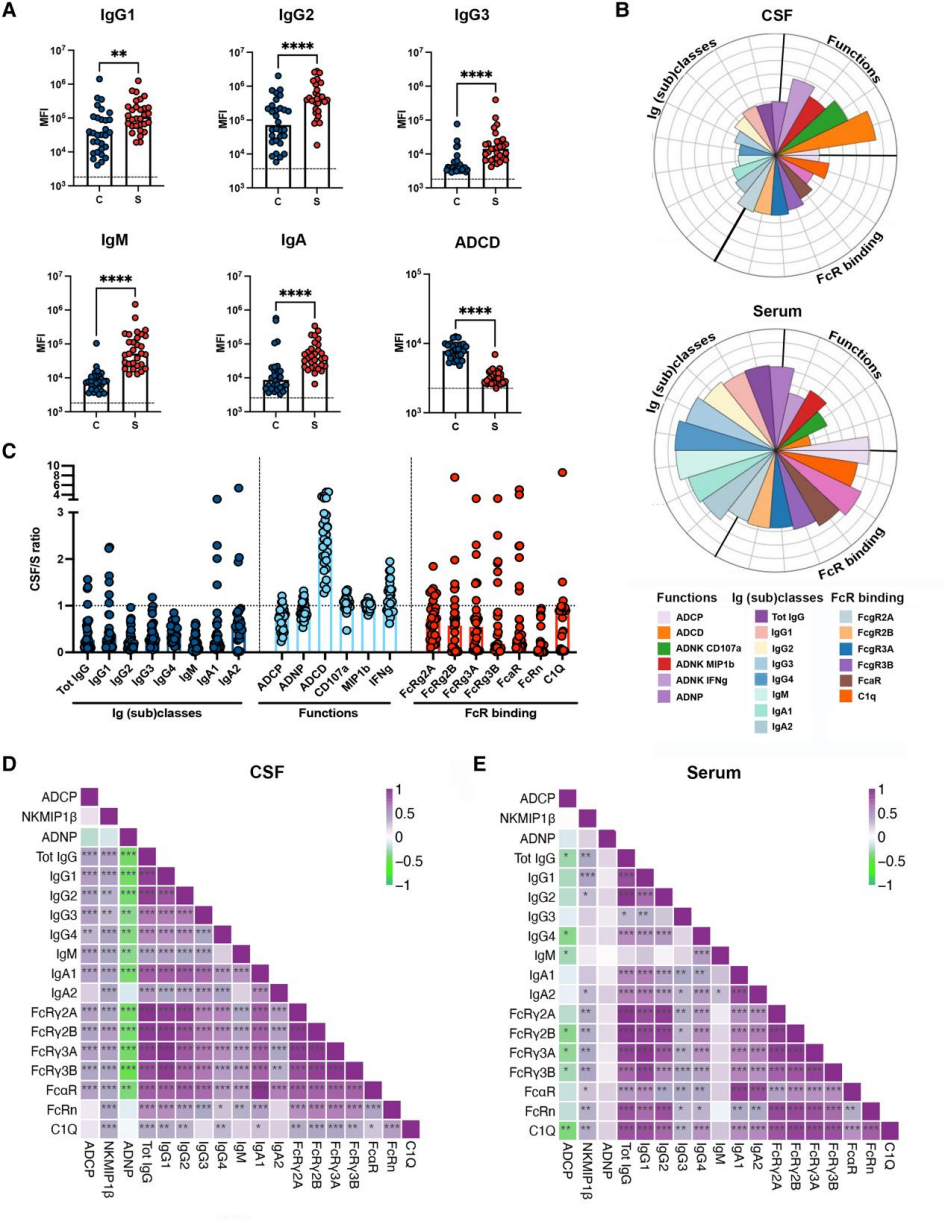
**Compartmentalization and coordination of the humoral response to *Mycobacterium tuberculosis* in serum and CSF from individuals with tuberculosis meningitis**. (**A**) Scatter-dot plot showing lipoarabinomannan (LAM)-specific antibody classes (IgG, IgM, IgA), subclasses (IgG1, IgG2, IgG3) and capacity to activate complement (ADCD) in the CSF (C = blue) and serum (S = red) from tuberculosis (TB) meningitis (*n* = 30). Each dot represents an individual. Bars represent median, dotted line indicates PBS level. Mann–Whitney statistics; **P < 0.01, ****P < 0.0001. (**B**) Flower plots showing LAM-specific IgG subclasses (1–4), IgM, IgA, Fc-receptors (FcRs: FcγR2A, FcγR2B, FcγR3A, FcγR3B and FcαR) and C1q binding, and antibody-mediated functions [antibody-dependent complement deposition (ADCD), antibody-dependent neutrophil phagocytosis (ADNP), THP-1 monocyte phagocytosis (antibody-dependent cellular phagocytosis, ADCP) and antibody-dependent natural killer cell activation (ADNK CD107a, ADNK MIP1β, ADNK IFNγ)] in serum and CSF of TB meningitis (*n* = 30). Each flower plot summarizes the data from the respective compartment and the length of each petal represents the average of the *z*-scored value for the indicated feature. (**C**) Scatter-dot plot showing CSF:serum ratios of LAM-specific IgG subclasses (1–4), IgM, IgA, binding to FcRs (FcγR2A, FcγR2B, FcγR3A, FcγR3B, FcαR) and C1q, and antibody-mediated functions [ADCD, ADNP, THP-1 monocyte phagocytosis (ADCP) and ADNK (ADNK CD107, IFNγ, MIP1β)], showing an enrichment in highly functional (and in particular ADCD-mediating) antibodies in CSF of TB meningitis (*n* = 30). Each dot represents an individual. Bars represent median. (**D** and **E**) Correlation matrix between LAM-specific antibody features [Ig classes (IgG, IgM, IgA), Ig subclasses (IgG1, IgG2, IgG3, IgG4, IgA), functions (ADCD, ADCP, ADNP, NK MIP1β) and binding to C1q and FcRs (FcγR2A, FcγR2B, FcγR3A, FcγR3B, FcαR)] showing higher humoral response coordination in CSF compared with serum of TB meningitis (*n* = 30). Correlation strength is proportional to colour intensity [ρ: from −1 = negative correlation (green) to 1 = positive correlation (purple)]. (Spearman’s correlation, Benjamini–Hochberg correction for multiple comparisons; *P < 0.05, **P < 0.01, ***P < 0.001).

We next aimed to determine whether CSF antibody responses differed from systemic responses elicited during TBM. Thus, we deeply profiled the *Mtb*-specific antibody response across the two compartments in our TBM cohort. Focusing on the immunodominant LAM-specific responses, we noted that a variety of LAM-specific IgG subclasses, IgM and IgA responses were detectable in the CSF (Fig. 2A). However, these levels were lower than those detected in the serum and were associated with a decreased capacity to bind to all Fc-receptors (FcγR2A, FcγR2B, FcγR3A, FcγR3B, FcαR) and C1q (Fig. 2B). Nevertheless, compared with serum responses, CSF responses exhibited an enhanced capacity to mediate complement activity (ADCD) and, to a lesser extent, NK cell activation (Fig. 2A and B). To confirm the differential enrichment of particular antibody subpopulations across the serum and CSF, we examined the LAM-specific CSF:serum ratios (Fig. 2C). While most humoral responses were enriched in the serum (lower ratios), the CSF exhibited a selective enrichment in highly functional antibodies, and in particular complement-activating antibodies. Similarly, antibodies targeting LAM and other *Mtb*-antigens demonstrated enhanced ratios for binding to C1q, the first component of the complement cascade,^26^ further supporting a selective enrichment of complement functions mediated by antibodies in the CSF of individuals with TBM (Fig. 2C and Supplementary Fig. 1).

To define differences in the humoral responses between compartments, we explored the overall architecture of the humoral immune response in each compartment by evaluating differences in coordination between LAM-specific antibody functions, isotypes, subclasses, and FcR binding. Enhanced overall coordination was observed in the CSF humoral immune responses compared with the serum (Fig. 2D and E). Specifically, the functional responses in the CSF were more tightly linked to different antibody subclasses/isotypes, and Fc-receptor binding profiles. These findings support the presence of different qualities of antibodies across compartments. Also, given the varied subclass and isotype profiles in the CSF, these data point more likely to a local (i.e. within the brain) production of humoral immune responses (rather than sieving of antibodies from the peripheral circulation across the blood–brain barrier), able to explore a broad array of antibody types, that may simply be programmed to elicit antibody effector functions, such as ADCD, that might be more effective within the CNS during *Mtb*-infection. Whether these results provide insights into the antibody functions that may be most relevant for CNS antimicrobial activity remains unclear.

### Association of *Mtb*-specific antibody profiles in serum (or CSF) and TBM disease severity or mortality

**Figure 3:**
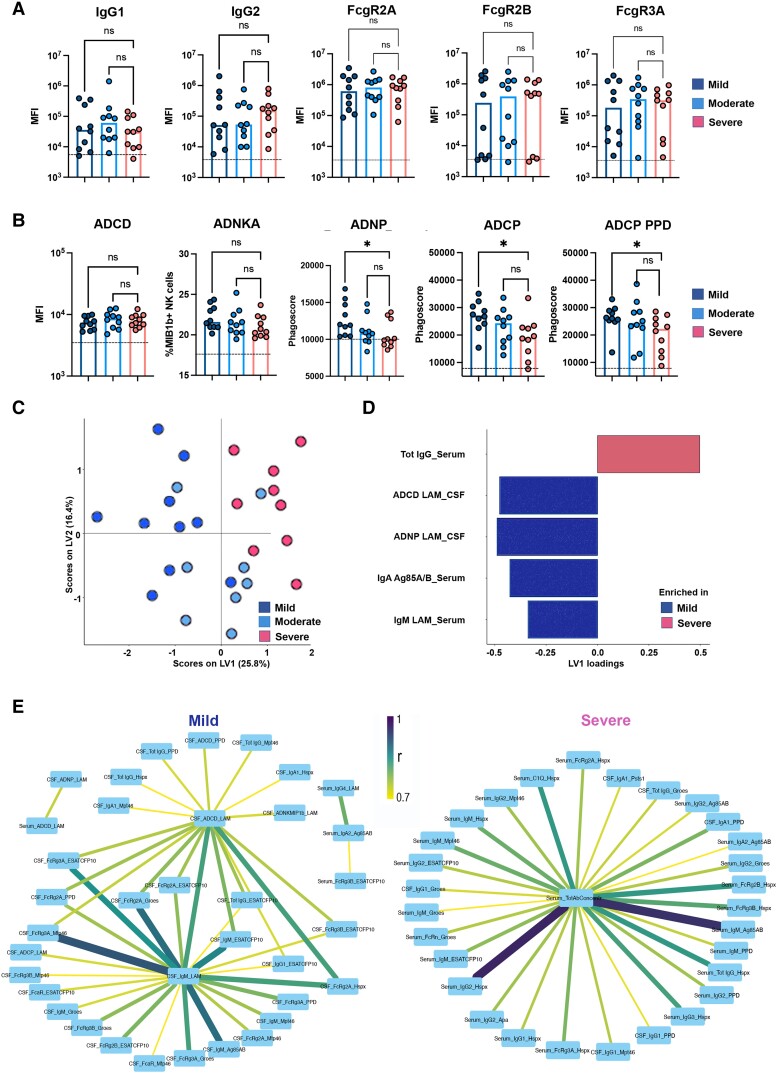
***Mtb*-antibody responses are associated with disease severity of tuberculosis meningitis.** (**A** and **B**) Scatter-dot plot showing lipoarabinomannan (LAM)-specific IgG1 and IgG2, binding to FcγR2A, FcγR2B, FcγR3A, antibody-mediated functions [THP-1 monocyte phagocytosis (antibody-dependent cellular phagocytosis, ADCP), antibody-dependent neutrophil phagocytosis (ADNP), antibody-dependent complement deposition (ADCD) and antibody-dependent natural killer cell activation (ADNKA)] and purified protein derivative (PPD)-specific ADCP in tuberculosis meningitis (TBM; *n* = 30) according to severity (mild = dark blue; moderate = light blue; severe = pink). Milder TBM disease showed higher levels of antibodies mediating ADCP (LAM- and PPD-specific) and ADNP (LAM-specific), despite having similar levels of IgG and FcγR binding. Each dot represents an individual. Bars represent median, dotted line indicates PBS level. Kruskal–Wallis statistics; **P < 0.01, ***P < 0.001, ****P < 0.0001. (**C**) Orthogonalized partial least squares regression (OPLSR) on least absolute shrinkage selection operator (LASSO)-selected features showing separation between antibody signatures depending on severity of TBM (mild = dark blue; moderate = light blue; severe = pink). Dots represent individual samples (*n* = 30) across all tested *Mtb* antigens (LAM, PPD, Ag85 A/B, ESAT/CFP10, PstS1, Hspx, Apa, Mtp46 and GroES). The performance of the algorithm was evaluated with R2 and Q2 metrics. R2 = 0.69 indicating a high predictive accuracy and Q2 = 0.55 indicating a good performance on test data in the cross-validation setting. (**D**) Bar graph shows LASSO-selected antibody features across *Mtb* antigens (LAM, PPD, Ag85 A/B, ESAT/CFP10, PstS1, Hspx, Apa, Mtp46, GroES) in TBM individuals (*n* = 30) according to severity of the disease (mild = blue; severe = pink), ranked by their variable importance in projection (VIP). Bars represent antibody features enriched in mild (blue) versus severe (pink). (**E**) Correlation network between LASSO-selected antibody features enriched in severe (Total IgG_Serum) or mild disease (ADCD_LAM_CSF; ADNP_LAM_CSF; IgA_Ag85_A/B Serum; IgM_LAM_Serum) and the remainder of features, including Ig classes and subclasses (Total IgG, IgG1, IgG2, IgG3, IgG4, IgM, IgA), Fc receptor (FcγR2A, FcγR2B, FcγR3A, FcγR3B, FcαR) and C1q binding, and functions (ADCD, ADCP, ADNP, NK CD107, NK IFNγ, NK MIP1β). Edge colour and size are proportional to the strength of correlation as shown in the colour bar. Only correlations with Spearman’s ρ > 0.7 and *P* < 0.01 are shown. Statistics: *Z*-scores, Spearman’s correlation, Benjamini–Hochberg correction for multiple comparisons.

To investigate whether *Mtb*-specific CSF-humoral profiles associate with TBM severity at presentation, we examined whether *Mtb*-specific antibody profiles differed across the three modified BMRC grades (Table 1). Antibody responses were compared across subjects classified into mild (grade 1), moderate (grade 2) and severe (grade 3) disease (Fig. 3). In the CSF, no significant univariate differences were noted in LAM-specific antibody titres or FcγR binding antibody levels (Fig. 3A). However, CSF LAM-specific antibodies had a greater propensity to activate cellular phagocytosis by monocytes and neutrophils (ADCP and ADNP) in individuals with mild disease compared with severe disease, as also observed for PPD-specific ADCP (Fig. 3B), pointing to a functional difference in *Mtb*-specific antibody quality across disease severity.

To further explore whether a specific multivariate signature, across all *Mtb* antigen-specific humoral immunity in the CSF and serum, was associated with differences in disease severity, we performed a LASSO to first reduce the number of antibody features to the minimal features that captured the overall variance across the cohort. These features were then used to quantify and visualize separation across the subjects using an OPLSR.^25^ Using serum and CSF profiles, we observed a clear separation across disease severity (Fig. 3C; model performance using *R2* = 0.69 and *Q2* = 0.55 metrics indicating high predictive accuracy and good performance in cross-validation). Specifically, just five of the total 189 features that we captured for each subject were sufficient to separate individuals into severe and mild disease, across LV1. Of these five features, four were enriched in mild disease, including LAM-specific CSF ADCD- and ADCP-mediating antibodies as well as serum LAM-IgM and Ag85A/B-IgA levels (Fig. 3D). Only one feature was enriched in severe disease, i.e. higher total IgG titres (not *Mtb*-specific), suggesting hypergammaglobulinaemia. Moreover, because LASSO conservatively down-selects all co-correlated features, with the goal of finding a minimal set of features that captures overall variation across populations, we next also examined the co-correlates of the LASSO selected features to gain additional insights into the biological differences across the groups. The networks (Fig. 3E) revealed that mild disease was associated with overall higher IgM titres to several *Mtb* antigens in the CSF, as well as a higher capacity to bind FcγRs and FcαR. In contrast, severe disease was associated with higher titres of *Mtb*-specific antibodies (targeting mainly Hspx and Mtp46) with elevated FcγR binding capacity in serum, rather than CSF. Taken together, these data argue for a role for highly functional complement-mediating IgM in the immune control of *Mtb* in the CSF, and suggest that hypergammaglobulinemia and overactive production of less-functional *Mtb*-specific humoral responses in serum, rather than in CSF, may be associated with increased disease severity, as has been reported for pulmonary TB disease.^9,15,27^

Finally, we aimed to explore the association between antibody feaures and mortality (Table 1). Although some antibody-mediated functions appeared to be significantly associated with death (e.g. ADNK_LAM), none of these features remained significant after correction for multiple comparisons. The failure to demonstrate an effect of antibody features on mortality might be related to the small sample size and relative rarity of the event (only 5 of 30 patients died), resulting in statistical underpower.

### *Mtb*-specific antibody profiles differ across TBM and pulmonary TB

**Figure 4:**
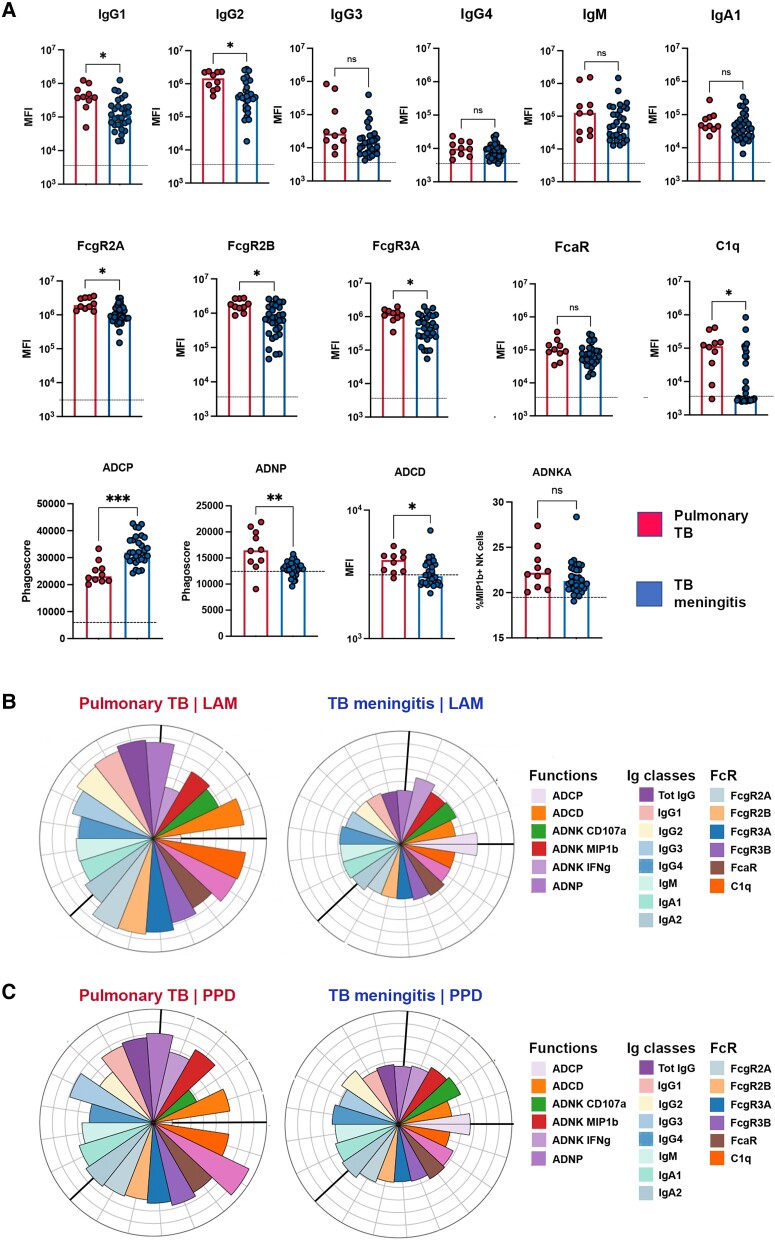
**Functional differences of lipoarabinomannan (LAM)-specific serum antibodies between tuberculosis (TB) meningitis and pulmonary TB.** (**A**) Scatter-dot plot showing LAM-specific Ig classes (IgG, IgM, IgA), subclasses (IgG1-4), Fc receptor (FcR; FcγR2A, FcγR2B, FcγR3A, FcγR3B, FcαR) and C1q binding, and antibody-mediated functions [complement deposition (antibody-dependent complement deposition, ADCD), neutrophil phagocytosis (antibody-dependent neutrophil phagocytosis, ADNP), THP-1 monocyte phagocytosis (antibody-dependent cell phagocytosis, ADCP) and natural killer (NK) cell activation (antibody-dependent NK activation, ADNKA)] in the serum of TB meningitis (blue, *n* = 30) compared pulmonary TB (red, *n* = 10). Compared with pulmonary TB (red), LAM-specific antibodies from TB meningitis (blue) show overall lower titres and FcR binding capacity but mediate higher ADCP. Each dot represents an individual. Bars represent median, dotted line indicates PBS level. Mann–Whitney statistics; **P < 0.01, ***P < 0.001, ****P < 0.0001. MFI = median fluorescence intensity. (**B** and **C**) Flower plots showing LAM- and purified protein derivative (PPD)-specific Ig classes (IgG, IgM, IgA), subclasses (IgG1–4), FcR (FcγR2A, FcγR2B, FcγR3A, FcγR3B, FcαR) and C1q binding, and antibody-mediated functions [ADCD, ADNP, THP-1 monocyte phagocytosis (ADCP) and ADNK (ADNK CD107a, ADNK MIP1β, ADNK IFNγ)] in serum from TB meningitis (*n* = 30) and pulmonary TB (*n* = 10). Each flower plot summarizes the data from the respective group and the length of each petal represents the average of the *z*-scored value for the indicated feature.

Given that TB largely manifests as a pulmonary infection, we next aimed to determine whether *Mtb*-specific antibody profiles in serum differ across individuals with TBM and pulmonary TB (clinical information provided in Table 1). At a univariate level, serum from pulmonary TB was marked by higher LAM-specific IgG1 and IgG2, with high capacity to bind to FcγR and C1q and to activate ADCD and ADNP (Fig. 4A). Similarly expanded antibody titres and FcgR binding capacity were also observed for other *Mtb*-antigen specific responses (Supplementary Fig. 2). Conversely, subjects with TBM exclusively exhibited an expansion of LAM-specific ADCP activity (Fig. 4A and B) as also observed for PPD-specific responses (Fig. 4C). These data point to distinct systemic antibody profiles that could differentiate TBM from individuals with pulmonary TB.

### Similar serum *Mtb*-specific antibody signatures between definite and probable TBM

**Figure 5:**
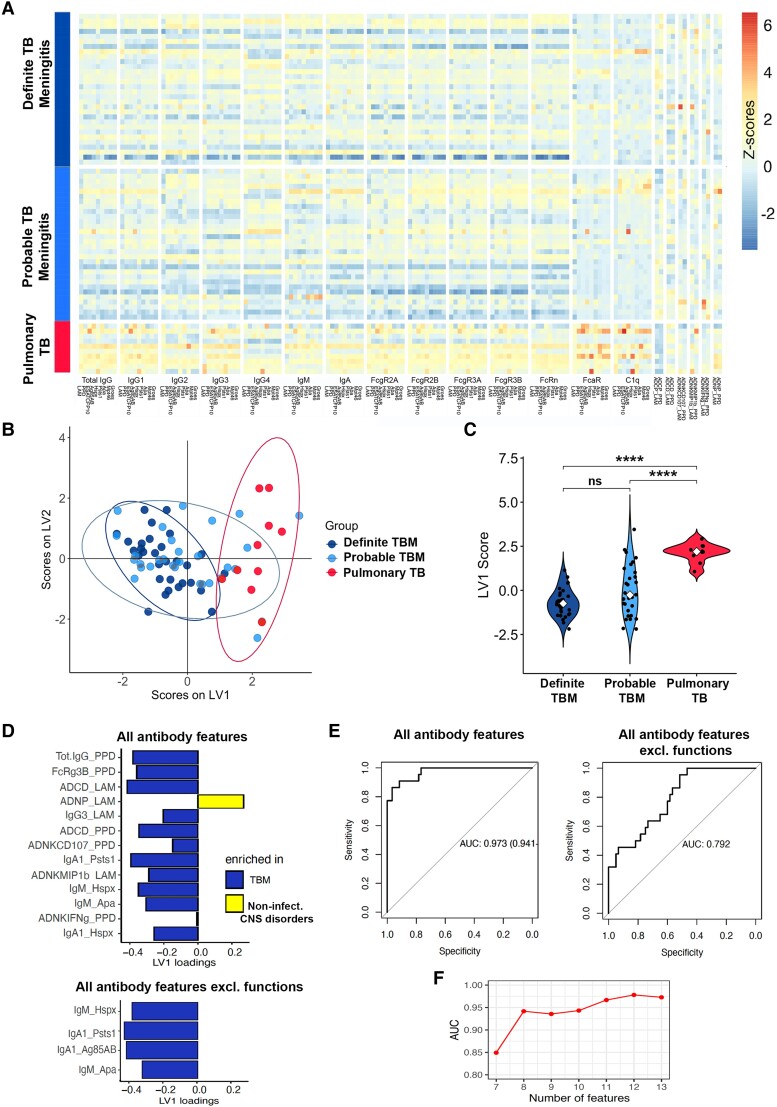
**Similar serum antibody signatures between definite and probable tuberculosis meningitis (TBM) and performance of a potential diagnostic test using CSF antibody signatures that distinguish TBM from non-infectious CNS disorders**. (**A**) Heatmap of Ig classes (IgG, IgM, IgA), binding to Fc receptors (FcRs; FcγR2A, FcγR2B, FcγR3A, FcγR3B, FcαR) and C1q, and antibody-mediated functions [complement deposition (antibody-dependent complement deposition, ADCD), neutrophil phagocytosis (antibody-dependent neutrophil phagocytosis, ADNP), THP-1 monocyte phagocytosis (antibody-dependent cell phagocytosis, ADCP) and natural killer (NK) cell activation (antibody-dependent NK activation, ADNKA; ADNK CD107, IFNg, MIP1b)], across all tested *Mtb* antigens [lipoarabinomannan (LAM), purified protein derivative (PPD), Ag85 A/B, Hspx, ESAT/CFP10, Psts1, Apa, Mtp46 and GroES] in definite (*n* = 30) and probable (*n* = 25) TBM, compared with pulmonary TB (*n* = 10). *Z*-scored values, colour-coded from dark blue (negative *z*-scores) to yellow and red (positive *z*-scores). (**B**) Partial least square discriminant analysis (PLSDA) on least absolute shrinkage selection operator (LASSO)-selected features showing separation between antibody signatures in pulmonary TB (red, *n* = 10) and probable TBM (light blue, *n* = 25). The graph has been overlapped with the PLSDA model showing separation between pulmonary TB (red) and definite TBM (dark blue, *n* = 30), to show the large overlap between probable and definite TBM individuals. Dots represent individual samples across all tested *Mtb* antigens (LAM, PPD, Ag85 A/B, ESAT/CFP10, Psts1, Hspx, Apa, Mtp46 and GroES). (**C**) Violin plots show latent variable 1 (LV1) scores from PLSDA model across pulmonary TB (red, *n* = 10) and definite (dark blue, *n* = 30) or probable (light blue, *n* = 25) TBM. Kruskal–Wallis statistics; ****P < 0.0001, ns = not significant. (**D**) Bar graph shows LASSO-selected antibody features across *Mtb* antigens (LAM, PPD, Ag85 A/B, ESAT/CFP10, PstS1, Hspx, Apa, Mtp46 and GroES) in TBM individuals (definite and probable) compared with non-infectious CNS disorders, ranked by their Variable Importance in Projection (VIP) scores. Bars represent antibody features enriched in TBM (blue) versus non-infectious CNS disorders (yellow) when considering all antibody features (*top*, 13 features selected) or all features except functions (*bottom*, four features selected). (**E**) Receiver operating characteristic (ROC) curves illustrating the performance, in terms of area under the curve (AUC) for sensitivity and specificity, of a potential diagnostic test discriminating TBM (definite and probable) from non-infectious CNS disorders, using the LASSO-selected features from **D** as biomarkers. The ROC curve using the 13 LASSO-selected features (from the ‘all antibody features’ model) shows excellent performance (AUC 0.97, *left*). The ROC curve using the four LASSO-selected features (from the ‘all antibody features except functions’ model) shows good performance (AUC 0.79, *right*). (**F**) AUC values of ROC curves from the ‘all antibody features’ model in **D** using seven antibody classes/subclasses and FcR-binding features, among the 13 LASSO-selected features, and adding the functions one by one in order of their VIP scores. In this model, adding the first function (ADCD_LAM) improves the AUC from 0.85 to 0.95, whereas adding the following five functions (ADNP_LAM; ADCD_PPD; ADNK_CD107a_PPD; ADNK_MIP1b_LAM; and ADNK_IFNg_PPD) results in only a minor change in the AUC (plateau).

Diagnosis of TBM is challenging because, clinically, it can mimic several other infectious and non-infectious neurological conditions. Also, the yield of *Mtb* cultures from CSF is suboptimal, leading to false negative results and delayed diagnosis. Thus, here we aimed to explore whether antibody responses were similar between definite, in which *Mtb* was identified in CSF and/or other body fluids, and probable TBM (clinical information provided in Table 1), in which *Mtb* was not identified. Interestingly, we observed that *Mtb*-specific antibody features were similar between these two TBM groups, and significantly differed from those with pulmonary TB (Fig. 5A). To further confirm similarities between antibody signatures in definite and probable TBM, we performed a PLSDA model to compare each group with pulmonary TB (Fig. 5B). Both models significantly differed from the antibody profiles in pulmonary TB, and no difference were observed between the TBM groups (Fig. 5C).

### Antibody signatures as diagnostic biomarkers for TBM compared with non-infectious CNS disorders

To explore these antibody features as biomarkers of TBM, we developed PLSDA-LASSO models and developed receiver operating characteristic (ROC) curves to explore the performance, in terms of sensitivity and specificity, of a potential diagnostic test in CSF discriminating TBM from non-infectious CNS disorders. A first model, using all antibody features, selected 13 features (Fig. 5D, top) and showed an outstanding discriminating performance [area under the curve (AUC) 0.97; Fig. 5E, left] between the two groups. Nevertheless, as antibody-mediated functions might not be easily performed as routine diagnostic tests (because of time consumption, cost and high biological variability), unlike the Luminex-beads based assays used for Ig classes and FcR-binding analyses, we built another model using all features except antibody-mediated functions. This model, in which as few as four features were sufficient to discriminate between TBM and non-infectious CNS disorders (Fig. 5D, bottom), showed a good discriminating performance (AUC 0.79; Fig. 5E, right). Finally, to understand the minimum number of antibody-mediated functions with best discrimination between the two groups, we examined the AUC of the ROC curves using the 7 Ig (sub)classes and FcR-binding LASSO-selected features (Fig. 5D) and adding one function at a time. Importantly, just by adding one function (ADCD_LAM), the test performance increased from 0.85 to 0.95, whereas it did not change substantially when sequentially adding five further functions (Fig. 5F). Overall, these results support a role for CSF antibody signatures as a potential diagnostic test to help clinicians differentiating between TBM (especially in those cases where *Mtb* cannot be confirmed) and non-infectious CNS disorders.

## Discussion

TBM is the most severe form of *Mtb* infection.^2^ To date, there is no vaccine that efficiently prevents TBM in adults, and treatment requires 9–12 months of multiple antibiotics, with risks of poor compliance and serious liver, renal and haematologic toxicities.^28^ Even when adequately treated, TBM continues to cause high mortality,^29^ and survivors are often left with life-long neurological sequelae.^4,30,31^ Host immune response is considered to play a major role in the pathology and outcome of TBM.^32^ Our study points to a potential role for immune mechanisms in contributing to TBM severity, and identifies CSF compartmentalized antibody signatures, in particular phagocytosis-mediating antibodies, as potentially protective from the development of more severe TBM. During pulmonary TB, *Mtb* growth is restrained through the formation of granulomas. This immune response seems to significantly differ from the one developing during TBM, where granulomas are more rarely formed and persistent immune activation often leads to neuronal injury.^32^ Here, we demonstrated functionally divergent humoral profiles, characterized by highly functional and FcγR binding antibodies in pulmonary TB, in contrast to attenuated profiles in TBM with selective enrichment of cellular phagocytosis functions (ADCP). Overall, these results reveal divergent humoral and innate immune profiles and potential mechanisms of anti-microbial control across sites of infection, and highlights the importance of compartment-specific immunity in the control of *Mtb*.

Although many patients present with TBM without radiologic evidence of previous pulmonary TB infection, the development of TBM is thought to begin with respiratory infection, followed by haematogenous spread to the CNS.^33^*Mtb* invasion of the CNS may occur through a ‘Trojan horse’ mechanism, where infected macrophages and neutrophils migrate across the blood–brain barrier and reach the brain parenchyma; alternatively, *Mtb* bacilli might gain access to the brain via infection of endothelial cells of the blood–brain barrier.^33^ Once in the brain, *Mtb* infection in the meninges or the subpial or subependymal spaces leads to development of tuberculous lesions (Rich foci).^7^ The rupture of these foci, with release of bacteria into the subarachnoid space, is believed to elicit a robust host immune response and the onset of meningitis. A thick inflammatory exudate around the basal meninges typically forms that causes many of the common TBM complications. These include obstruction of the CSF flow causing hydrocephalus and raised intracranial pressure or the occlusion of cerebral arteries, leading to brain ischaemia, stroke and cranial nerve palsies.^33^ Thus, *Mtb*-induced neuroinflammation, rather than direct bacterial-related mechanisms, is thought to be the main responsible of brain injury.^4^ This is supported by the improved survival in individuals with TBM treated with adjunctive corticosteroids and by the association between decreased CSF cytokines and better outcome.^34^ Our study further supports the involvement of immune mechanisms in determining brain disease and defines an important role for CSF humoral responses in protecting from severe TBM, with enrichment of LAM- and PPD-specific phagocytosis- and complement-mediating antibodies in milder disease.

Antibodies in the CSF are usually derived from blood circulation and are transported across the blood–brain barrier through an FcRn-mediated mechanism.^35^ However, in disease settings, antibodies can be produced locally by B-cells that were recruited into the brain and matured to antibody-producing plasma cells. Given the preference of FcRn for IgGs,^36,37^ and particularly IgG1 and IgG3, the broad repertoire of antibody subclasses and isotypes we observed in the CSF argue for antibody production within the brain. This CSF immune compartmentalization is in line with previous studies showing distinct transcriptomic patterns of immune activation in the CSF of TBM.^38^ These studies also demonstrated correlation between levels of neuroinflammation and neuronal damage, further supporting the idea of compartmentalized immune response playing a detrimental role in the pathogenesis of TBM.^38^ Interestingly, RNA sequencing studies^38^ also showed hyperactivation of inflammasome and reduced T-cell activation, which is substantially different from the immune responses observed in pulmonary TB, where initial contention of *Mtb* growth by innate immune cells is followed by recruitment of T cells, which play a major role in the formation of granulomas and control of latency/activation cycle during respiratory infection. Our study supports this concept of distinct immune responses based on the site of infection, and expands on this by highlighting unique systemic humoral signatures that distinguish pulmonary TB from TBM. Despite overall attenuated antibody profiles, TBM showed an enrichment of cellular phagocytosis-mediating antibodies in serum, and complement activating antibodies in the CSF, that indicate a remarkable selectivity of antibodies with specific effector functions not only locally, within the brain, but also peripherally, during the maturation of the immune response against *Mtb*. Importantly, we demonstrated that similar antibody profiles are also found in probable TBM and defined the CSF antibody features that most distinguish TBM from other non-infectious CNS disorders. Overall, our data support that antibody signatures might be used as a complement of diagnosis in TBM, especially in those cases where *Mtb* cannot be confirmed, as demonstrated by the high performance (in terms of sensitivity and specificity) of a potential diagnostic test using a combination of these features (with or without antibody-mediated functions).

Additional studies, across different geographical sites, will likely be required to validate the value of *Mtb*-specific antibody responses as markers of TBM and confirm the use of these features as potential diagnostic test to facilitate the differential diagnosis with other infectious and non-infectious CNS disorders. Moreover, future research is needed to confirm the role of antibody-mediated phagocytosis and complement activation in the protection from *Mtb* burden in the brain, which will pave the way to develop new therapeutic strategies using rationally designed monoclonals that leverage the local immune response by promoting protective innate immune functions and preventing brain damage.

## Supplementary Material

awae066_Supplementary_Data

## Data Availability

The data that support the findings of this study are available from the corresponding author, upon reasonable request.
