# Supplementary material for: Distinctive antibody responses to Mycobacterium tuberculosis in pulmonary and brain infection
Source: Brain. 2024 Mar 5;147(9):3247–60. doi: 10.1093/brain/awae066 (PMC11370789; doi:10.1093/brain/awae066)
Supplement: awae066_Supplementary_Data [file awae066_supplementary_data.pdf]

Supplemental material

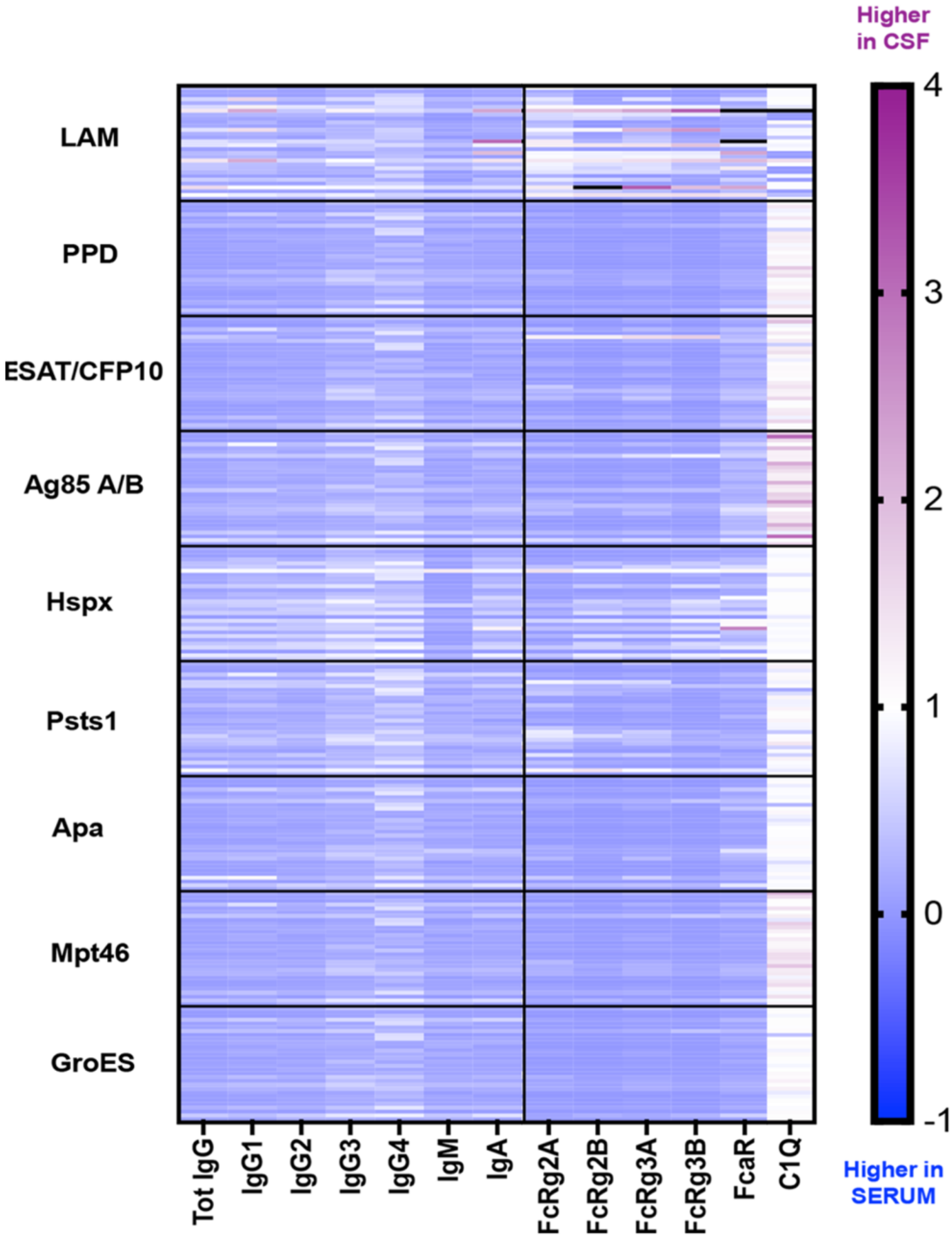

Supplemental Fig. 1. **Enrichment of C1q binding antibodies in CSF of TBM.** Heatmap showing CSF:serum ratios of IgG subclasses (1–4), IgM, IgA, and binding to FcRs (FcγR2A, FcγR2B, FcγR3A, FcγR3B, FcαR) and C1q for each antigenic target (LAM, PPD, ESAT/CFP10, Ag85 A/B, Hspx, PstS1, Apa, Mpt46, GroES). This shows an overall higher titer and FcR bidning capacity by serum antibodies, in contrast to a relative enrichment of C1q binding antibodies in CSF. CSF:serum ratios are color coded (>1 in purple, indicating enrichment in CSF, <1 in blue, indicating enrichment in serum).

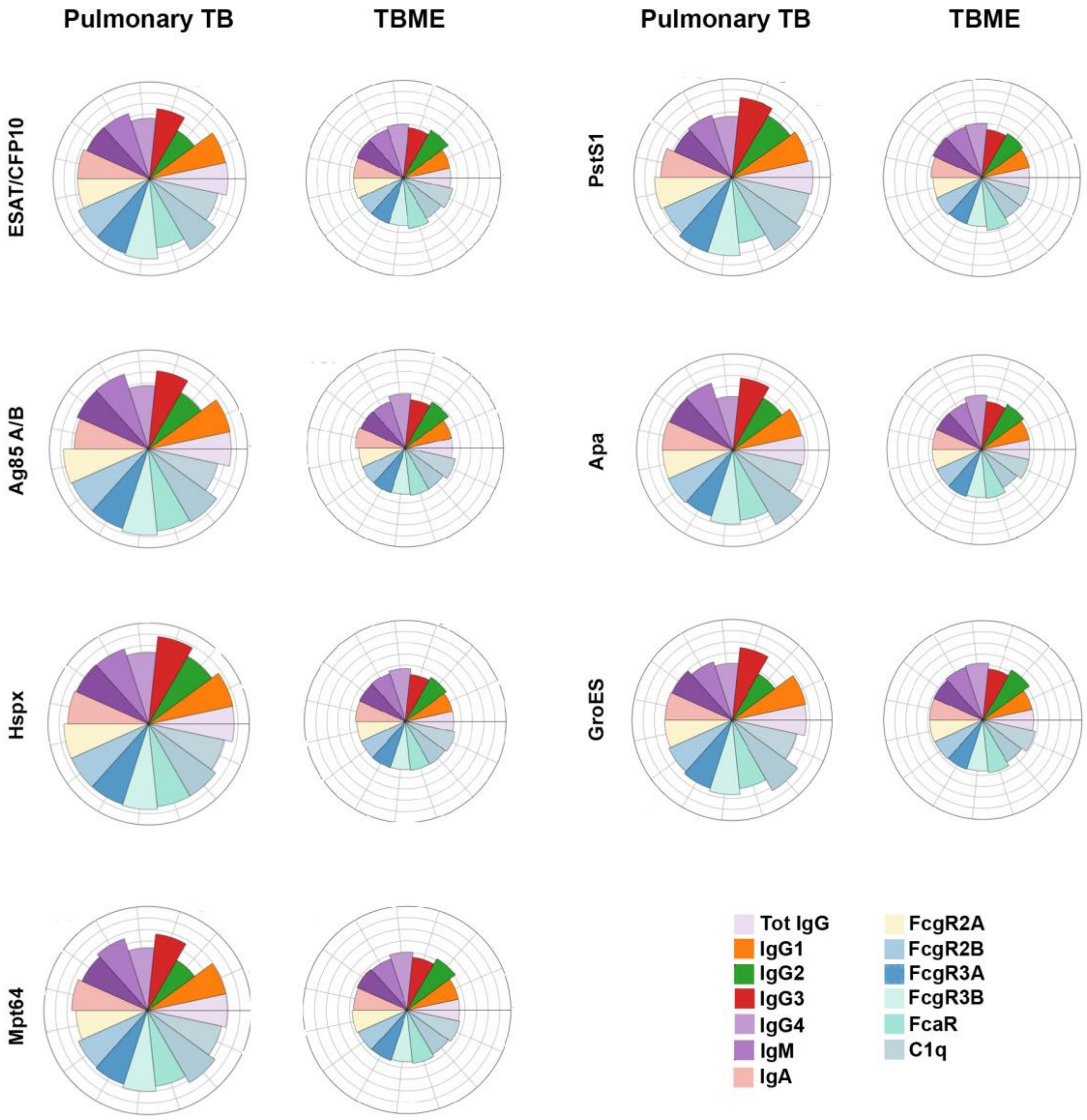

Supplemental Fig. 2. Pulmonary TB shows *Mtb*-specific antibodies with higher titers and FcR binding capacity than TB meningitis (TBM). Flower plots showing IgG subclasses (1–4), IgM, IgA, binding to FcRs (FcγR2A, FcγR2B, FcγR3A, FcγR3B, FcαR) and C1q in pulmonary TB versus TBME, across different antigen specificities (ESAT/CFP10, Ag85 A/B, Hspx, Psts1, Apa, Mpt46, GroES). Each flower plot summarizes the data from the respective group and the length of each petal represents the average of the Z-scored value for the indicated feature.
